# Association between Exposure to Alcohol’s Harm to Others and Health-Related Quality of Life in Korean Adults: A Nationwide Population-Based Study

**DOI:** 10.3390/ijerph18052714

**Published:** 2021-03-08

**Authors:** Mi-Jung Eum, Min-Jung Choi

**Affiliations:** 1 Department of Nursing Science, Kyungbuk College, 77 Daehakro, Yeongju-si 36133, Gyeongbuk, Korea; emj44@hanmail.net; 2 College of Nursing, The Catholic University of Korea, 222 Banpo-daero, Seocho-gu, Seoul 06591, Korea

**Keywords:** population, exposure to alcohol’s harm to others, health-related quality of life

## Abstract

This study aimed to investigate the association between exposure to alcohol’s harm to others (AHTO) and health-related quality of life HRQoL using a nationally representative sample. Data from 2016–2018 of 17,346 South Korean individuals aged ≥19 years were obtained from the Korea National Health and Nutrition Examination Survey. HRQoL was evaluated using the European Quality of Life–5 Dimensions (EQ-5D) index. A significant positive association was found between exposure to AHTO and lower EQ-5D scores (*p* = 0.022). In the final model of multiple regression analysis, participants’ HRQoL decreased by 0.932 points when exposed to AHTO (R^2^ = 36.5%, *p* < 0.001). In addition, the exposure to AHTO group had significantly higher odds ratios (OR) for pain/discomfort (OR: 1.42, 95% CI: 1.15–1.75) and anxiety/depression (OR: 1.55, 95% CI: 1.68–2.80) compared to the non-AHTO group. Further studies are required for intervention when exposed to reduce the pain/discomfort and anxiety/depression in AHTO victims as well as to reduce the incidence of AHTO.

## 1. Introduction

As of 2017, the per capita annual alcohol consumption among South Korean adults was 8.7 L, which is similar to the average of countries in the Organization for Economic Co-operation and Development (OECD; 8.9 L) [1]. However, as of 2018, in South Korea, the high-risk drinking and binge drinking rates were 14.7% [2] and 38.9% [3], respectively, markedly exceeding the rate of 7% and 16.4% surveyed by the Centers for Disease Control and Prevention [4]. Heavy alcohol consumption and binge drinking adversely affect the physical and mental health of not only the drinker [5] but also of people around them (via secondhand effects) [6]. The adverse effects imposed on health, safety, and quality of life of other people due to an individual’s alcohol consumption is known as alcohol’s harm to others (AHTO). A 2015 survey of AHTO in the United States found that 20% of 8750 adult participants suffered from AHTO [7]. Similarly, a 2017 report of 3000 Korean adults reported that 24.1% of participants experienced AHTO [8]. Therefore, secondhand drinking, as with secondhand smoking, is an important issue to be addressed in public health.

AHTO includes traffic accidents related to drunk driving, physical and mental abuse, family conflicts, and financial troubles [7]. A recent study reported that exposure to AHTO is associated with age [9], sex [10], race [11], marital status [9], income [12], education level [9], family history of alcoholism [13], the individual’s own drinking habits [14], and public health policies [12]. Furthermore, another study reported that AHTO affects well-being and mental health, including anxiety and depression [15].

Health-related quality of life (HRQoL) represents an individual’s perceived well-being in the physical, mental, and social domains of health [16]. The European Quality of Life–5 Dimensions (EQ-5D) questionnaire is a particularly useful tool in for measuring HRQoL in the general adult population, owing to its simple and organized layout [17].

Past studies have analyzed the association between exposure to AHTO and HRQoL and mental well-being [6,18,19]; however, these only assessed participants’ general characteristics, socioeconomic status, and frequency of exposure to an excessive drinker [13], without considering other factors related to HRQoL, such as activity limitation [20], body mass index [21], physical activity [22], smoking habits [23], and history of chronic diseases [24,25].

Thus, this study aims to analyze the association between exposure to AHTO and HRQoL among Korean adults, considering their socio-demographic characteristics, health behaviors, and comorbidities, based on nationally representative and standardized data.

## 2. Materials and Methods

### 2.1. Survey Overview and Study Population

The Korean National Health and Nutrition Examination Survey (KNHANES) is an annual national cross-sectional survey conducted by the Korea Disease Control and Prevention Agency (KCDA) [26]. This study used data from the years 2016–2018. To ensure a nationally representative sample, the KNHANES sampling frame uses data from the most recent population and housing census, which was based on a two-stage stratified cluster sampling method across districts and households. The KNHANES consists of a health interview, a health examination, and a nutrition survey. The health interview and examination are carried out using standardized procedures in mobile examination centers.

**Figure 1:**
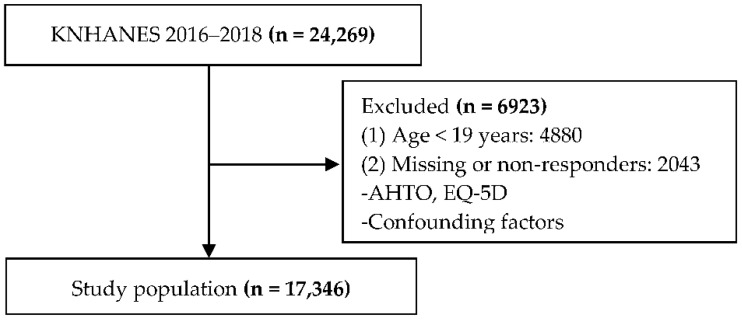
Flow diagram illustrating participant selection.

This study initially collected data from 24,269 individuals. However, individuals who did not meet our inclusion criteria, because they were aged <19 years (*n* = 4880) or had missing data (*n* = 2043), were excluded. Therefore, a final sample comprising 17,346 individuals was used for statistical analysis (Figure 1).

### 2.2. Assessment of the Exposure to Alcohol’s Harms to Others

To assess participants’ experiences of exposure to AHTO, we gathered data from the following item from the 2016 to 2017 KNHANES: “Have you ever experienced turmoil, assault, sexual harassment, accidents during work or daily life (injuries), or drunk driving accidents due to the alcohol drinking of others in the past 12 months?” Participants answered “yes” or “no.” In the 2018 KNHANES, participants were instructed to specify what they had experienced: (1) turmoil, (2) assault, (3) sexual harassment, (4) accidents during work or daily life (injuries), and/or (5) drunk driving accidents. Participants were allowed to select more than one of these experiences; individuals who responded “yes” to any of these were assigned to the exposure to AHTO group.

### 2.3. Assessment of HRQoL

HRQoL was evaluated using the Korean version of the European Quality of Life-5 Dimensions (EQ-5D) scale developed by the EuroQol Group (The EuroQol Group, Rotterdam, Zuid-Holland, The Netherlands) [17]. The EQ-5D scale measures five dimensions of HRQoL: mobility, self-care, usual activities, pain/discomfort, and anxiety/depression. Each dimension is assessed using a single question with three possible answers. “No problems” was scored as 0 points, whereas the responses “some problems” and “extreme problems” were scored as 1 point.

Total EQ-5D index was calculated using the correction formula derived from the quality weight-scoring system used by the KCDA for the Korean population [27]. In this study, the EQ-5D index was multiplied by 100 for readability. The Korean version of the EQ-5D scale has been previously evaluated for validity and reliability [28].

### 2.4. Potential Confounding Variables

Participants were classified into the following age groups: 19–29, 30–39, 40–49, 50–59, and ≥60 years old. Education level was classified as follows: elementary school or below, middle school, high school, and university or higher. Household income was divided into four quartiles calculated as the equalized income according to the sample household income in the KNHANES. Marital status was classified as with spouse, without spouse, and unmarried. Participants who answered “yes” to the economic activities item were considered employed.

Smoking status was defined as never-smokers, former smokers (had smoked ≥100 cigarettes during their lifetime but were not smoking currently), and current smokers (had smoked ≥100 cigarettes and were still smoking). Alcohol consumption was classified as never-drinkers, moderate drinkers (who have drunk alcohol but who drink less than heavy drinkers), and heavy drinkers (consumed alcohol at least twice a week, with ≥7 servings/sitting [61 g] for men and ≥5 servings/sitting [40 g] for women) [29]. Physical activity was defined as the practice of moderate-intensity activity for at least 150 min a week, high-intensity activity for 75 min a week, or a combination of moderate and high-intensity activity (one minute of high-intensity activity was converted to two minutes of moderate-intensity activity) [30].

Participants who answered “yes” to activity limitations reported feeling restricted in daily living and social activities due to health problems or physical/mental disorders. Stress level was classified as low (low/very low) or high (high/very high). Subjective health status was categorized as good (good/very good), average, and bad (poor/very poor). BMI was classified into two groups: obese (≥25 kg/m^2^) and non-obese (<25 kg/m^2^) [31]. Medical history detailed whether they had ever been diagnosed by a physician with any of the following diseases: cancer (stomach, liver, colon, breast, cervix, lung, or thyroid cancer), circulatory diseases (hypertension, hyperlipidemia, stroke, myocardial infarction, or angina), musculoskeletal disorders (osteoarthritis, rheumatoid arthritis, or osteoporosis), and endocrine disease (diabetes mellitus).

### 2.5. Statistical Analysis

SPSS Statistics software, version 23 (IBM Corp., Armonk, NY, USA) was used for all statistical analyses to account for the complex sampling design. In this study, complex samples were analyzed by applying the weights suggested by KCDA. Data are presented as percentages (standard error (SE)) for categorical variables and as mean ± SE for continuous variables. A comparison of participants according to their exposure to AHTO and characteristics (including demographic and socioeconomic characteristics, health behaviors, health conditions, and EQ-5D scores) was performed using the F-test for continuous variables in several groups and a Pearson chi-square test for categorical variables as a test of homogeneity. This study evaluated the association between exposure to AHTO and HRQoL (as participants’ EQ-5D scores) using multiple linear regression analyses. The association between exposure to AHTO and the five dimensions of the EQ-5D was analyzed by multiple logistic regression analyses. The odds ratios (ORs) and 95% confidence intervals (CIs) of reporting problems in any of the sub-dimensions of the EQ-5D were calculated in the exposure to AHTO group, compared with the non-AHTO group. Model I was adjusted for age and sex. Model II was adjusted for age, sex, education level, marital status, household income, employment status, smoking status, alcohol consumption, physical activity, and BMI. Model III was additionally adjusted for stress level, subjective health status, activity limitation, and medical history. Statistical significance (*p* < 0.05) was defined using a two-tailed test.

### 2.6. Ethical Considerations

For the KNHANES, the KCDA obtained informed consent from all participants, whose personal data were anonymized. This study was conducted with the approval of the Institutional Review Board of the Catholic University of Korea (IRB-MC20ZASI016; approval date: 15 December 2020).

## 3. Results

### 3.1. Participants’ Characteristics Based on Their Exposure to AHTO

Table 1 presents participants’ characteristics and their exposure to AHTO. Demographic data, health behaviors, clinical characteristics, and EQ-5D scores varied significantly depending on their exposure to AHTO.

Exposure to AHTO was higher among men (4.8%) and those who were young (19–29 years old), had a higher education level, were unmarried, and were unemployed (*p* < 0.05). The proportion of current smokers and problem drinkers was higher in the AHTO group. Furthermore, participants with a higher level of stress and poorer subjective health were more likely to be exposed to AHTO.

The AHTO group had a significantly lower score in the EQ-5D sub-dimension of pain/discomfort (*p* = 0.001) and anxiety/depression (*p* < 0.001) compared with the non-AHTO group.

### 3.2. Multiple Linear Regression Analysis Regarding Associations between the Exposure to AHTO and HRQoL

Table 2 shows the results of multiple linear regression analysis of the association between exposure to AHTO and EQ-5D scores. Significantly positive associations were found between exposure to AHTO and EQ-5D scores, after adjusting for confounding variables (all *p*’s < 0.001). In Model I, R^2^ was 11.2% and EQ-5D scores decreased by 1.813 points in the adjusted EQ-5D scores when individuals reported exposure to AHTO. These associations persisted after adjustment for socioeconomic and health behavior confounding factors in Model II. Even after adjusting all confounding variables in Model III, R^2^ was 36.5%, while the adjusted EQ-5D scores of participants in the AHTO group were 0.932 points lower than those of participants in the non-AHTO group.

### 3.3. Associations between the Exposure to AHTO and Each Dimension of the EQ-5D

Table 3 shows the multiple logistic regression analysis of the association between exposure to AHTO and each dimension of the EQ-5D. In Model I, the ORs for reporting problems in the sub-dimensions of mobility (OR: 1.49; 95% CI: 1.11–2.01), usual activities (OR: 1.58; 95% CI: 1.08–2.30), pain/discomfort (OR: 1.69; 95% CI: 1.36–2.08), and anxiety/depression (OR: 2.19; 95% CI: 1.69–2.83) were higher in the AHTO group compared with the non-AHTO group. In Model II, the ORs for reporting problems were higher in the AHTO group in all sub-dimensions when compared with the non-AHTO group (all *p*’s < 0.05). After adjusting for all potential confounding variables (Model III), the AHTO group had significantly higher ORs for reporting problems related to pain/discomfort (OR: 1.42; 95% CI: 1.15–1.75; *p* < 0.001) and anxiety/depression (OR: 1.55; 95% CI: 1.17–2.05; *p* < 0.003).

## 4. Discussion

This study analyzed the association between exposure to AHTO and HRQoL using a dataset representative of the South Korean population. Of the 17,346 participants, 4.4% reported exposure to AHTO. Even after adjusting for socio-demographic factors, health behaviors, and health conditions, AHTO and low HRQoL were correlated, while the percentage of people who reported pain/discomfort and anxiety/depression was higher among people exposed to AHTO.

In this study, young male and unmarried individuals were associated with exposure to AHTO. Previous studies have also reported that having these characteristics increases the risk of exposure to AHTO. However, some studies have reported that those aged 35–44 [32], female [12,33], or married [32] experienced AHTO more frequently. However, in most studies, young individuals were exposed to AHTO at a high rate, similar to these results [33,34]. Based on previous studies suggesting that individuals over the age of 40 place themselves less frequently in environments that may expose them to AHTO, younger adults would be likely to place themselves more frequently in risky drinking situations with the potential for violence and injury [12]. These findings suggest that young, male, and unmarried people should be aware of the risk of both direct and indirect harm from alcohol.

In addition, the results related to socioeconomic status (SES) were mixed. In this study, exposure to AHTO was higher among the unemployed, but there was no association with household income. Similarly, previous studies reported no significant relationship between exposure to AHTO and low economic status [14,19]. These findings suggest that high exposure to AHTO is associated with behavioral factors (e.g., the individual’s involvement in high-risk drinking), health-related factors (e.g., stress level and subjective health status), and environmental factors (e.g., the presence of heavy drinkers around the individual) as well as low SES. Therefore, further research examining the effects of the presence of heavy drinkers around participants is required to conduct a broader exploration of the relationship between SES and AHTO.

A key question in this study was whether exposure to AHTO is associated with HRQoL even after adjusting for all confounding variables. There may be a bias reflected in the outcome of the relationship between AHTO and HRQoL because the EQ-5D index was assessed using current status, but exposure to AHTO was examined for the previous 12 months. Nevertheless, a conservative interpretation of the study’s results supports the idea that exposure to AHTO is associated with a low HRQoL (Table 2). This finding is consistent with previous studies in which people with exposure to AHTO who interacted with heavy drinkers had a lower EQ-5D index and personal well-being index [18,32,35]. These results indicate that AHTO should be considered in public health interventions related to alcohol-related harms.

The main strength of this study is that the analysis was performed using a large sample size and considering potential new covariates. People who are exposed to AHTO reported pain/discomfort and anxiety/depression more frequently compared to participants in the non-AHTO group. These results are consistent with previous findings [6,7,34,36]. People who were exposed to AHTO felt unsafe [34], stress, self-sacrifice, worry, fear, and anxiety [36], all of which contribute to a negative effect on HRQoL. According to a previous study, not only strangers but also friends and cohabitants are highly likely to be perpetrators of AHTO [37]; furthermore, if the perpetrator is an acquaintance, victims’ experience of AHTO, such as prolonged disturbances, violence, sexual assault, and physical injury, may cause stress and strains comparable to providing long-term care for a person in a poor health condition [37]. Specifically, women whose husbands have alcohol problems often suffer from depression, anxiety, and lower satisfaction with life [36]. Previous studies reported that people who were exposed to AHTO have more problems in the sub-dimension of mobility and usual activity [32]. However, in this study, there was no difference in these sub-dimensions after adjusting for activity limitation and comorbidities (Table 3). These results indicate that, even in physically healthy adults, exposure to AHTO has a negative effect on HRQoL. Previous studies have also reported that husbands’ alcohol drinking had a greater effect on their wives’ mental health compared to their physical health [6]. Therefore, proactive efforts are required to reduce pain/discomfort and anxiety/depression in individuals exposed to AHTO even in physically healthy individuals. However, the interpretation of these results should take into account that there was an absence of questions about the relationship between the perpetrator and the victim and the duration of exposure to AHTO.

The World Health Organization (WHO) mentioned AHTO as a focus of concern in the global strategy to reduce the harmful use of alcohol [1]. Nevertheless, the South Korean society has a relatively lenient culture toward AHTO [38]. Thus, in addition to punishment and education for individuals regarding the HRQoL effects of AHTO, community-led efforts are required to improve the social and physical environment that are similar to measures protecting against the hazards of passive smoking.

This study has a few limitations. First, this study used self-reported questionnaires; therefore, results may have recall bias. Second, the KNHANES is a useful resource to obtain a large-scale, nationally representative dataset, but the causality among the variables cannot be established. Thus, we recommend that long-term prospective studies be conducted to investigate the effects of AHTO on HRQoL. Third, in this study, we could not identify the frequency, level of severity, and type of exposure to AHTO as well as whether the participants’ cohabitants were problematic drinkers. Fourth, while this study adjusted for confounding factors identified in past studies, other important confounders may also be present.

## 5. Conclusions

The results of this study show that exposure to AHTO is an important predictor of HRQoL. In addition, this study highlights the need to consider pain/discomfort and anxiety/depression in people with a history of exposure to AHTO, even in physically healthy adults. It is believed that these findings will be helpful in determining the direction of appropriate interventions to improve the HRQoL of people exposed to AHTO. Moreover, these results will be valuable as the rationale for policy enforcement on preventing AHTO for public health.

## Figures and Tables

**Table 1:** Characteristics of study participants and exposure to AHTO.

Variables	Category	Non-AHTO (*n* = 16,651)	AHTO (*n* = 695)	Statistics ^b^	*p*
		% (SE) or Mean ± SE ^a^			
Sex	Male	95.2 (0.3)	4.8 (0.3)	7.769	0.016
	Female	96.1 (0.2)	3.9 (0.2)		
Age (years)	19–29	93.4 (0.6)	6.6 (0.6)	71.885	<0.001
	30–39	95.6 (0.4)	4.4 (0.4)		
	40–49	95.8 (0.4)	4.2 (0.4)		
	50–59	95.2 (0.4)	4.8 (0.4)		
	≥60	97.5 (0.2)	2.5 (0.2)		
Education level	≤Elementary	97.9 (0.3)	2.1 (0.3)	40.585	<0.001
	Middle school	96.1 (0.6)	3.9 (0.6)		
	High school	95.0 (0.4)	5.0 (0.4)		
	≥College	95.3 (0.3)	4.7 (0.3)		
Household income	Lowest	95.9 (0.5)	4.1 (0.5)	0.776	0.917
	Lower intermediate	95.5 (0.4)	4.5 (0.4)		
	Higher intermediate	95.6 (0.4)	4.4 (0.4)		
	Highest	95.6 (0.4)	4.4 (0.4)		
Marital status	Married (with spouse)	96.0 (0.2)	4.0 (0.2)	51.212	<0.001
	Married (without spouse)	97.3 (0.4)	2.7 (0.4)		
	Unmarried	93.8 (0.5)	6.2 (0.5)		
Employment status	Employed	96.4 (0.3)	3.6 (0.3)	14.259	0.002
	Unemployed	95.6 (0.3)	4.4 (0.3)		
Smoking	Current smoker	94.0 (0.5)	6.0 (0.5)	32.663	<0.001
	Former smoker	95.7 (0.4)	4.3 (0.4)		
	Never smoker	96.2 (0.2)	3.8 (0.2)		
Alcohol consumption	Heavy	93.2 (0.7)	6.8 (0.7)	38.936	<0.001
	Moderate	96.0 (0.3)	4.0 (0.3)		
	None	96.0 (0.3)	4.0 (0.3)		
Physical activity	Yes	95.3 (0.3)	4.7 (0.3)	4.623	0.090
	No	95.9 (0.2)	4.1 (0.2)		
Stress level	Much	93.0 (0.5)	7.0 (0.5)	114.643	<0.001
	Little	96.7 (0.2)	3.3 (0.2)		
Subjective health status	Good	96.5 (0.3)	3.5 (0.3)	28.060	<0.001
	Average	95.7 (0.3)	4.3 (0.3)		
	Bad	94.0 (0.5)	6.0 (0.5)		
Body mass index	≥25 kg/m^2^	95.7 (0.3)	4.3 (0.3)	0.104	0.778
	<25 kg/m^2^	95.6 (0.2)	4.4 (0.2)		
Activity limitation	Yes	95.2 (0.7)	4.8 (0.7)	0.667	0.492
	No	95.7 (0.2)	4.3 (0.2)		
Cancer	Yes	96.5 (0.8)	3.5 (0.8)	1.255	0.308
	No	95.6 (0.2)	4.4 (0.2)		
Circulatory disease	Yes	96.5 (0.3)	3.5 (0.3)	11.240	0.008
	No	95.3 (0.2)	4.7 (0.2)		
Musculoskeletal disorders	Yes	97.3 (0.4)	2.7 (0.4)	12.898	0.001
	No	95.4 (0.2)	4.6 (0.2)		
Endocrine diseases	Yes	95.6 (0.5)	4.4 (0.5)	0.003	0.960
	No	95.6 (0.2)	4.4 (0.2)		
EQ-5D scores ^c^		95.57 ± 0.102	94.60 ± 0.417	5.299	0.022
Mobility	Problem	95.8 (0.5)	4.2 (0.5)	0.099	0.780
	No	95.6 (0.2)	4.4 (0.2)		
Self-care	Problem	95.7 (1.1)	4.3 (1.1)	0.001	0.982
	No	95.6 (0.2)	4.4 (0.2)		
Usual activities	Problem	95.2 (0.8)	4.8 (0.8)	0.503	0.547
	No	95.7 (0.2)	4.3 (0.2)		
Pain/Discomfort	Problem	94.4 (0.5)	5.6 (0.5)	16.593	0.001
	No	96.0 (0.2)	4.0 (0.2)		
Anxiety/Depression	Problem	92.3 (0.9)	7.7 (0.9)	41.936	<0.001
	No	95.9 (0.2)	4.1 (0.2)		

^a^ Values are presented as the percentages (SE) or mean ± SE. ^b^ Continuous variables are F values, and categorical variables are chi-square values for homogeneity. ^c^ EQ-5D scores are multiplied by 100.

**Table 2:** Multiple regression analysis between exposure to AHTO and EQ-5D scores.

Variables	Model I			Model II			Model III		
	B	SE	*p*	B	SE	*p*	B	SE	*p*
AHTO (ref. Non-AHTO)	−1.813	0.412	<0.001	−1.888	0.388	<0.001	−0.932	0.322	0.004
R^2^	0.112			0.189			0.365		
F	171.543 (<0.001)			74.688 (<0.001)			103.919 (<0.001)		

Model I was adjusted for sex and age; Model II was adjusted for Model I plus education level, marital status, household income, employment status, smoking, alcohol consumption, physical activity, and body mass index; Model III was adjusted for Model II plus stress level, subjective health status, activity limitation, and comorbidities.

**Table 3:** Odds ratio (95% CI) of having problems in each dimension of the EQ-5D, based on the experiences of AHTO.

Variables	Non-AHTO (*n* = 16,651)	AHTO (*n* = 695)	*p*
**Mobility**			
Model I	Reference	1.49 (1.11–2.01)	0.009
Model II	Reference	1.61 (1.20–2.15)	0.001
Model III	Reference	1.32 (0.97–1.79)	0.080
**Self-care**			
Model I	Reference	1.48 (0.88–2.48)	0.138
Model II	Reference	1.69 (1.01–2.82)	0.044
Model III	Reference	1.35 (0.80–2.31)	0.264
**Usual activities**			
Model I	Reference	1.58 (1.08–2.30)	0.018
Model II	Reference	1.74 (1.19–2.55)	0.004
Model III	Reference	1.34 (0.89–2.03)	0.161
**Pain/Discomfort**			
Model I	Reference	1.69 (1.36–2.08)	<0.001
Model II	Reference	1.71 (1.39–2.10)	<0.001
Model III	Reference	1.42 (1.15–1.75)	0.001
**Anxiety/Depression**			
Model I	Reference	2.19 (1.69–2.83)	<0.001
Model II	Reference	2.17 (1.68–2.80)	<0.001
Model III	Reference	1.55 (1.17–2.05)	0.003

Model I was adjusted for sex and age; Model II was adjusted for Model I plus education level, marital status, household income, employment status, smoking, alcohol consumption, physical activity, and body mass index; Model III was adjusted for Model II plus stress level, subjective health status, activity limitation, and comorbidities.

## Data Availability

The data that support the findings of this study are available from the Korean National Health and Nutrition Examination Survey at https://knhanes.cdc.go.kr/knhanes/eng/index.do (accessed on 21 December 2020).
